# Social identity threat is related to ethnic minority adolescents’ social approach motivation towards classmates via reduced sense of belonging

**DOI:** 10.1007/s11218-023-09800-3

**Published:** 2023-06-13

**Authors:** Laura Froehlich, Nathalie Bick, Jana Nikitin, Sarah E. Martiny

**Affiliations:** 1 FernUniversität in Hagen, Hagen, Germany; 2 University of Vienna, Vienna, Austria; 3 UiT The Arctic University of Norway, Tromsø, Norway

**Keywords:** Ethnic minority students, Social identity threat, Sense of belonging, Social approach motivation, Multiple social identities

## Abstract

The integration of ethnic minority youth can only be successful if they are motivated to establish and maintain social relationships in important institutions such as school. At the same time, worries about negative stereotypes about one’s ethnic group can undermine ethnic minority students’ motivation to approach others. In the present study, we tested whether social identity threat predicts ethnic minority adolescents’ social approach motivation via reduced sense of belonging. We also examined whether multiple social identities (i.e., high endorsement of ethnic and national idenitiy) buffer against the negative effects of social identity threat. In a sample of 426 ethnic minority students from 36 9^th^ -grade classes in Germany, social identity threat was indirectly related to social approach motivation via reduced sense of belonging to the school and class. The interplay of students’ ethnic and national identity moderated the relationship of social identity threat and sense of belonging. The relationship was particularly negative for students who endorsed either ethnic or national identity. However, it was less negative for students with integrated multiple social identities and non-significant for students who identified neither with the ethnic nor the national group. Results generalized for social approach motivation towards ethnic majority and minority classmates. These patterns were only found for social approach motivation in face-to-face contact situations, but not in online situations. We discuss these findings in light of the literature on social identity threat and multiple social identities. Practical implications include measures to foster students’ sense of belonging and to reduce social identity threat.

## Introduction

Germany is a multicultural country and ethnic heterogeneity is present in the educational system as approximately 40% of children and adolescents below the age of 15 have a migration background (Statistisches Bundesamt, 2022). Full integration of immigrants is often defined as equal chances of participation in societal domains like education and the labor market irrespective of ethnicity (e.g., Esser 2001). However, some ethnic groups are not fully integrated in Germany and perform worse than natives (e.g., Weis et al., 2020). This is particularly true for students with ethnic origins from Turkey, South-East Europe, and Northern Africa (e.g., Stanat et al., 2019). Ethnic achievement gaps could be partly explained by segregated peer networks in school and classroom contexts, as performance and learning is limited if instrumental help-seeking relationships are mainly focused on shared (ethnic) group membership rather than who is the most competent classmate (e.g., Zander et al., 2019). Negative stereotypes about the competence of various ethnic groups are widespread in Germany (Froehlich & Schulte, 2019). Members of ethnic minority groups might worry about confirming these negative stereotypes in the school context (i.e., social identity threat) and thus be less motivated to establish and maintain relationships with peers. We examine this hypothesis in a sample of ethnic minority adolescents in Germany. Furthermore, we investigate the role of ethnic and national identity in this context. Results of the present study inform about how stereotypes influence interethnic relations in the classroom and thus contribute to ethnic inequalities in educational integration.

### Consequences of social identity threat for ethnic minority students’ performance and social relationships

Research on social identity threat (e.g., Aronson & McGlone 2009) has shown that negative stereotypes about ethnic minority students’ competence can threaten their positive social identity (Steele et al., 2002). Studies investigating stereotype threat as a specific form of social identity threat found that the activation of negative stereotypes impairs standardized test performance in experimental settings (e.g., Steele & Aronson 1995). Stereotype threat contributes to the explanation of ethnic minority students’ academic underperformance (e.g., Appel et al., 2015; Froehlich et al., 2022; Weber et al., 2018). In addition to these well-documented performance-reducing effects of stereotype threat, recent studies also showed consequences of social identity threat in the social domain. Experimental and correlational studies with different negatively stereotyped groups (i.e., female and immigrant university students as well as older employees) showed that social identity threat is associated with social approach motivation (Froehlich et al., 2023; Martiny & Nikitin, 2019; Rahn et al., 2021). Social approach motivation is the motivation to initiate and maintain positive peer relationships (Nikitin & Schoch, 2021). It forms the basis for positive social contact (Gable & Berkman, 2008; Nikitin & Schoch, 2021), and is associated with less loneliness and more relationship satisfaction (Gable, 2008; Nikitin & Freund, 2017).

Verkuyten et al. (2019) proposed a model based on identity process theory to explain the relation between ethnic-racial discrimination and ethnic minority students’ academic (dis)engagement. The authors assumed that ethnic-racial discrimination invokes social identity threat. The model proposes that ethnic-racial discrimination threatens the satisfaction of the social identity needs of belonging, esteem, and control, which in turn relate to cognitive, affective, and behavioral aspects of academic engagement and performance. Applied to the current research, it can be assumed that social identity threat compromises the satisfaction of ethnic minority students’ need to belong, as negative stereotypes convey the message that minority students do not fully belong to the academic context (Walton & Cohen, 2007). If the satisfaction of the need of social belonging or relatedness is threatened, this reduces academic motivation and engagement (e.g., Deci & Ryan 1991). The current research extends this proposed mechanism from performance-related measures of academic engagement to the social domain and assumes that a threatened satisfaction of the need to belong also impairs social approach motivation.

It has indeed been empirically shown that the relation of social identity threat and social approach motivation is mediated by a reduced sense of belonging to the stereotyped domain (Froehlich et al., 2023; Martiny & Nikitin, 2019; Rahn et al., 2021). Impaired sense of belonging has been identified as a consequence of social identity threat (e.g., Walton & Cohen 2007, 2011). The indirect effect of social identity threat on social approach motivation via sense of belonging means that social identity threat is associated not only with a reduced sense of “fitting in” the relevant domain (e.g., the academic domain), but also with less motivation to form social connections in this domain. In a sample of ethnic minority university students, lower approach motivation, in turn, predicted lower intention to participate in social events to meet fellow students, indicating the important role of social approach motivation in the social integration of ethnic minority students (Froehlich et al., 2023). Being less motivated to form relationships for example by attending fewer social events might eventually lead to social isolation, which is in turn negatively associated with academic performance (e.g., Raabe 2019).

It should be noted that previous results on the direction of the direct effect of social identity threat on social approach motivation were mixed. Three studies with female university students (Martiny & Nikitin, 2019) showed a negative effect of experimentally manipulated social identity threat on social approach motivation, whereas two studies with ethnic minority university students (Froehlich et al., 2023) showed a positive relation of measured social identity threat and social approach motivation. Five studies with older employees in the workplace (Rahn et al., 2021) showed sometimes positive, but sometimes non-significant or negative relations for manipulated and measured social identity threat. Thus, the directionality of the direct effect seems to vary with target groups (gender, ethnicity, age), contexts (university, workplace), and methods (experiments, surveys). However, the negative indirect effect via reduced sense of belonging has been consistently shown to explain part of the association of social identity threat and social approach motivation. Based on these theoretical considerations and empirical evidence, we focus on the indirect effect and hypothesize that ethnic minority adolescents who worry about confirming negative stereotypes about their ethnic group in the school context feel less belonging to the academic context and are in turn less motivated to socially approach their classmates.

Most previous studies have assessed sense of belonging to the superordinate academic institution (i.e., the university; e.g., Martiny & Nikitin 2019), and some studies have assessed it at a lower institutional level (e.g., the computer science department; Walton & Cohen 2007). However, few studies include measures of sense of belonging at different institutional levels (Freeman et al., 2007). In the present research, we extend this earlier research by assessing students’ sense of belonging to the general academic institution (i.e., school) and on two further levels: the classroom and the teachers. We do so for two reasons. First, our study focuses on German secondary schools where adolescents are grouped in fixed classes over several years. Therefore, we assume that students form academic social relationships mainly with their classmates (but see Leszczensky & Pink (2015) for evidence of social networks on the grade level). Consequently, our measure of social approach motivation is directed towards peers in the classroom. We use sense of belonging to the classroom as the main mediating variable. As previous research also investigated the relation of school diversity policies and sense of belonging (e.g., Schachner et al., 2019), we also assess sense of belonging to school as the superordinate institution. Second, teachers in Germany predominantly belong to the ethnic majority group (Statistisches Bundesamt, 2010, 2019). As teachers evaluate students’ performance, they might be perceived by ethnic minority students as a source of social identity threat (Shapiro & Neuberg, 2007). Therefore, we additionally assess perceived acceptance by teachers as an alternative mediator of the relationship between social identity threat and social approach motivation.

### Generalizability or context-specificity of social approach motivation

Previous research has mainly investigated generalized social approach motivation towards peers. It is therefore an open question whether the indirect relationship between social identity threat and social approach motivation via reduced belonging is generalizable or specific to certain groups and contexts. The present research focuses on two major contextual dimensions: (a) interactions with ethnic minority vs. majority members (i.e., group membership) and (b) face-to-face vs. online interactions (i.e., mode of communication). Concerning group membership, studies on ethnic homophily have shown that people tend to befriend others who are culturally similar to themselves (e.g., Leszczensky & Stark 2020). This tendency is exacerbated by ethnic minority status: Friendship networks of minority students who identified with their ethnic group were particularly ethnically segregated (i.e., comprised more of ethnic ingroup members than majority group members; e.g., Jugert et al., 2018; Zander & Hannover, 2013). The rejection-identification model (Branscombe et al. 1999) describes that negative consequences associated with being the target of stereotypes and prejudice can be alleviated by an increased ethnic identification. It might thus be assumed that social identity threat particularly impairs minority students’ motivation to approach ethnic *majority* students (i.e., who are associated with being the predominant source of the widespread negative stereotype about the ethnic minority group). Concerning motivation to approach ethnic *minority* members, two opposing predictions can be made. On the one hand, the negative effect of social identity threat might not extend to ethnic minority members, as minority members turn towards ethnic ingroup members in the face of possible social rejection by majority members (e.g. Branscombe et al., 1999). Thus, in line with ethnic homophily, effects of social identity threat on social approach motivation might be specific to ethnic majority but not minority members. On the other hand, there might be a generalized effect of social identity threat on social approach motivation encompassing both ethnic majority and minority members, as stronger social identity threat is associated with a general feeling of “not fitting in” to the academic context as a whole (e.g., Walton & Cohen 2007). Social identity threat can also involve ingroup members as a source of the threat (i.e., concerns of confirming by one’s own behavior that the stereotype is true in the minds of ingroup members; Shapiro & Neuberg 2007). Therefore, the negative effect might extend to all peers in the academic context irrespective of their group membership.

Concerning the mode of communication, we explore whether social approach motivation is impaired both in face-to-face and online contact situations. Adolescents do not only interact face-to-face in their classrooms or schools, but also online, for instance via messengers or social media. Virtual peer interactions have increased in recent years due to technological developments and the COVID-19 pandemic (e.g., Hamilton et al., 2022; Twenge et al., 2019). Adolescents’ online and offline social networks are largely overlapping and a major function of online interactions is to maintain or expand offline social relationships (e.g., Liu et al., 2016; Reich et al., 2012). Consequently, social networks are not only ethnically segregated in offline face-to-face contact situations, but also in online situations (e.g., Dekker et al., 2015; Li et al., 2019). As online interactions with peers can constitute important resources for academic learning (Li et al., 2019), reduced social approach motivation in online contact situations might further impair the social relationships of ethnic minority students. The current research thus explores whether the relation of social identity threat and social approach motivation can be generalized to face-to-face and online contact situations.

### Multiple social identities and consequences of social identity threat for social relationships

Ethnic minority members usually endorse multiple social identities. That is, they can simultaneously identify with the host society (national identity) and their ethnic group of origin (ethnic identity; Fleischmann & Verkuyten 2016). Research on bicultural identity integration (e.g., Benet-Martínez & Haritatos 2005) has shown that integrated multiple identities are usually associated with higher sociocultural and academic engagement (Stogianni et al., 2021). However, they can be experienced either as harmonious and overlapping or as conflictual and compartmentalized. Fleischmann and Phalet (2016, p. 455) interpreted positive associations between social identities as compatibility in that identities “mutually reinforce one another”. Negative associations were interpreted as identity conflict (i.e., “some degree of tension” between identities in that the more individuals endorse one identity, the less they endorse the other); whereas non-significant correlations were interpreted as compartmentalization in that variation in one identity is “dissociated” or “decoupled” from variation in the other identity (Fleischmann & Phalet, 2016). The extent to which ethnic minority members are able to integrate their ethnic and national identities to harmonious and overlapping dual identities depends on individual as well as contextual factors (e.g., societal ideologies of assimilation vs. multiculturalism; Benet‐Martínez, 2012).

Froehlich et al. (2023) investigated how the interplay of ethnic and national identity moderates the relationship between social identity threat and sense of belonging. They argued that integrated multiple social identities (i.e., strong ethnic and strong national identity) act as a buffer (Kiang et al., 2008) against the negative consequences of social identity threat for social relationships. This should be the case because a simultaneous strong identification with both the minority and the majority group offers possibilities to approach members of both groups as potential friends. In line with this, results showed that higher social identity threat was not significantly related to social approach motivation via lower sense of belonging for students who strongly identified with both the ethnic and the national group (Froehlich et al., 2023). We aim to replicate this interaction effect and explore whether it is consistently found with the different measures of sense of belonging and social approach motivation included in the current study.

### The present research

The present research aims at replicating the findings on consequences of social identity threat for social approach motivation via reduced sense of belonging and the interplay with multiple social identities previously shown in adult samples with a sample of ethnic minority adolescents. We focus on adolescents for two main reasons. First, in this age group, social relationships are crucial for identity development and academic performance (e.g., Ragelienė 2016; Rodkin & Ryan, 2012) and thus reduced social approach motivation might diminish ethnic minority students’ educational integration. Second, we focus on secondary rather than tertiary education as the proportion of students with migration background is lower in tertiary compared to secondary education (Statistisches Bundesamt, 2022) as less selection effects have taken place before secondary school graduation.

We investigate the following pre-registered hypotheses: Sense of belonging mediates the relationship between social identity threat and social approach motivation (H1). The interaction of ethnic and national identity moderates the relationship of social identity threat and sense of belonging. The relationship is non-significant for students with a strong ethnic and a strong national identity (H2). We explore whether social approach motivation differs towards ethnic minority vs. majority members and in face-to-face vs. online contact situations. We explore to which extent sense of belonging to the class and the school, or perceived acceptance by teachers play a mediating role in the relationship between social identity threat and social approach motivation. Because we assess social approach motivation towards peer students within the classroom, we expect that sense of belonging to the class plays a stronger mediating role than sense of belonging to the school or perceived acceptance by teachers. The pre-registration, materials, data, and analysis scripts are available on the Open Science Framework [https://osf.io/v7tez/].

## Method

### Participants

A Monte Carlo simulation-based power analysis (Wang & Rhemtulla, 2021) based on effect sizes from Froehlich et al. (2023) with a power of 0.99 for the indirect effect (H1) and a power of 0.80 for the moderated indirect effect (H2) resulted in a required sample size of at least 319 ethnic minority students. We recruited 36 9^th^ grade classes from middle-track secondary schools (Real- / Gesamt-/ Sekundarschule) in ethnically heterogeneous urban regions in Germany. This resulted in an achieved total sample size of *N* = 710 students. Of those, *n* = 435 students indicated identifying with an ethnic minority and *n* = 275 students identified as German only. The final sample of ethnic minority students consisted of 426 students after applying the following pre-registered exclusion criteria: participants who did not consent or indicated that their data should not be used for scientific purposes (*n* = 4), and participants who left more than 50% of the questionnaire unanswered (*n* = 1). Furthermore, we excluded students who filled out the questionnaire for ethnic minority students but indicated “German” as their cultural group of origin (*n* = 2; exclusion criterion not pre-registered) or who fell outside of the usual age range of 9^th^ grade students by indicating that they were below 14 years (*n* = 1) or above 18 years (*n* = 1). This exclusion criterion was not pre-registered, but applied to ensure that the sample consisted of students who had reached the minimum required age to consent to study participation and who were in the developmental period of adolescence.

In the final sample of ethnic minority students, 213 students (50%) indicated identifying as male and 203 students (48%) as female (other: *n* = 8, 2%, *n* = 2 missing). Age ranged between 14 and 17 years (*M* = 15.05, *SD* = 0.78). The majority of students reported speaking both German and another language with their family (German: 76%, other: 89%) and friends (German: 98%, other: 57%). The largest ethnic groups were Turkish (*n* = 115, 27%), Polish (*n* = 48, 11%), Italian (*n* = 28, 7%), Moroccan (*n* = 22, 5%), Syrian (*n* = 17, 4%), Kosovan (*n* = 15, 4%), and Greek (*n* = 12, 3%). The other groups (i.e., Iranian, Serbian, Romanian, Iraqi, Bulgarian) were endorsed with lower frequencies. The category “other” was chosen by *n* = 155 (36%) of the sample. These participants reported origins from Arabic countries and ethnicities (e.g., Egypt, Afghanistan, Lebanon, Tunisia, Kurdish, Persian), different countries of the former UDSSR (e.g., Russia, Ukraine, Kazakhstan, Lithuania), or the Balkans (e.g., Albania, Bosnia, Serbia, Montenegro), other Western European countries (e.g., France, Portugal), or countries from Asia (e.g., Korea, Philippines, Thailand, Vietnam) and Africa (e.g., Ghana, Nigeria, Somalia). A classification of countries based on Froehlich and Schulte (2019) showed that 63% of countries of origin were negatively stereotyped as low on competence in the German context.

### Procedure

The study was approved by the first author’s institutional ethics commission and by the data protection officer. It adhered to regulations of the German General Data Protection Regulation and research ethics guidelines of the American Psychological Association. Parents were informed about data protection and the aims and procedure of the study and were given the opportunity to opt out of their children participating. Data were collected in Spring 2022 in classrooms with a teacher and a member of the research team present. Materials were cross-sectional paper-pencil questionnaires (at one school, the study was conducted online without a member of the research team present in a classroom on individual computers). The survey was administered in German language. Items not available in German were translated by the project team.

Students were informed about data protection and the aims and procedure of the study and gave their written consent for participation. Then they were instructed to choose one of two questionnaire versions. One version was for students who solely identified with Germans and the other version was for students who identified with an ethnic minority (i.e., identifying with a non-German ethnic group exclusively or in combination with the German group; for detailed instructions, see codebook on the OSF). The current research focused on data collected from students who completed the version for ethnic minorities. First, students indicated their ethnic group of origin and completed measures of ethnic, national, and dual identity. Then they completed measures of social identity threat, sense of belonging, social approach motivation, and demographic information. Finally, participants were given the opportunity to consent to their data being used for scientific purposes. After questionnaire completion, students were debriefed about the research project by the experimenter. The study was conducted within one school period (45 min). Classes received 150€ for their class fund as compensation for participation (on average 7.60€ per student).

### Materials

All measures, with the exception of demographics, were assessed using a 5-point Likert scale ranging from 1 = do not agree to 5 = completely agree. *Culture of origin and multiple social identities* were measured as follows: Participants were provided with a definition of culture of origin (Maehler, 2019), highlighting that culture pertains to traditions and values (e.g., music, food, clothing, values, religion) of the country their parents or grandparents were born in. Participants’ culture of origin was assessed with “which culture of origin best describes you?” and the most frequent ethno-cultural minority groups in Germany displayed as checkboxes (Maehler, 2019). We used the German version of the Multigroup Ethnic and National Identity Measure (MENI) including six items each to measure *ethnic identity* (e.g., “I feel a strong attachment to my culture of origin ”, Cronbach’s α = 0.83) and *national identity* (e.g., “I feel a strong attachment to German culture ”, α = 0.88; Maehler 2019). We additionally included a single-item measure of *dual identity* (“I feel German-[ethnic group]”; Martiny et al., 2017).

*Social identity threat* was assessed with four items by Martiny and Nikitin (2019) adapted to the school context (e.g., “I am worried that I might confirm stereotypes about the abilities of my cultural group of origin at school”, α = 0.81).

*Sense of belonging* was assessed with items adapted from Good et al. (2012) separately for belonging to school and class (subscales membership and acceptance, 4 items each, e.g., “At my school/in my class, I feel accepted”; “I feel like I belong at school/in my class”, school: α = 0.94, class: α = 0.97). Perceived acceptance by teachers was assessed with 4 items from the acceptance subscale (e.g., “I feel accepted by my teachers”; α = 0.93).

*Social approach motivation* was assessed with four items each separately for different ethnic groups in the classroom and in face-to-face as well as online contact situations. First, it was explained to students that contact to other students can take place face-to-face (directly talking in person in the classroom or the schoolyard) or online (via messengers or social media). Items were assessed separately for social approach motivation in face-to-face and online situations. Moreover, items were assessed separately regarding ethnic majority classmates, classmates of their own cultural group of origin (if present in their classroom), and non-German classmates with another cultural group of origin (e.g., “I am trying to establish and deepen my relationships with German students/ students of my own culture of origin/ students from other cultures of origin (online)”, 0.91 < α < 0.95). Items were adapted from Elliot et al. (2006).

*Demographics.* Participants completed measures of gender (male/ female/ other), age (years), as well as languages spoken with family and friends.

*Additional variables.* As an additional control variable, we categorized the countries of origin according to whether they are negatively stereotyped in the German context (i.e., low competence and low/moderate warmth: Turkey, Arabic and African countries, the Balkans) vs. not negatively stereotyped based on Froehlich and Schulte (2019; for detailed coding of countries of origin, see analysis code “syntax to paper” on the OSF).^1^

### Statistical analyses

Data preparation and exploratory descriptive analyses were conducted in SPSS 28. Multi-item constructs (i.e., ethnic identity, national identity, sense of belonging, and social approach motivation) were aggregated to scales. Pre-registered hypotheses were tested with path modeling in Mplus 8.8. Due to the clustered data structure (students nested in classrooms), we inspected intra-class correlation coefficients for all variables. ICCs were low (0.04 > ICC > 0.08) and the Design Effect was below 2 in all cases. Therefore, we report parsimonious models not accounting for the nested data structure. Results were unchanged in robustness checks controlling for the clustered data structure. The frequency of missing values was below 3% for all variables, therefore we used listwise deletion in SPSS (Lüdtke et al., 2007) and Full Information Maximum Likelihood (FIML) estimation in Mplus.

## Results

### Preliminary analyses

Descriptive statistics and bivariate correlations are depicted in Table 1. Exploratory investigation of mean values of multiple social identities showed that ethnic identity (*M* = 3.90, *SD* = 0.85) was endorsed more strongly than national identity (*M* = 2.70, *SD* = 1.01; *t*(424) = 19.42, *p* < .001, *d* = 0.94 95% CI [0.83; 1.06]) and dual identity (*M* = 3.47; *SD* = 1.26; *t*(381) = 5.33, *p* < .001, *d* = 0.27, 95% CI [0.17; 0.37]). Dual identity was endorsed more strongly than national identity (*t*(382) = 11.45, *p* < .001, *d* = 0.58, 95% CI [0.48; 0.69]). Exploratory investigation of the interrelations of multiple social identities showed that ethnic identity was not significantly associated with both national and dual identity, whereas national and dual identity were positively associated.

**Table 1:** Descriptive Statistics and Bivariate Correlations (*r*, [95% CI])

	*M* (*SD*)	(1)	(2)	(3)	(4)	(5)	(6)	(7)	(8)	(9)	(10)	(11)
(1) Ethnic Identity	3.90 (0.85)	-										
(2) National Identity	2.70 (1.01)	.07 [-.03; .16]	-									
(3) Dual Identity	3.47 (1.27)	-.07 [-.17; .03]	.38^***^ [.29; .46]	-								
(4) Social Identity Threat	2.01 (1.00)	.14^**^ [.05; .23]	.11^*^ [.01; .20]	.03 [-.07; .13]	-							
(5) Sense of Belonging to Class	3.91 (1.09)	.21^***^ [.12; .30]	.08 [-.02; .17]	.05 [-.06; .15]	-.18^***^ [-.27; -.08]	-						
(6) Sense of Belonging to School	3.78 (1.01)	.21^***^ [.11; .29]	.13^**^ [.03; .22]	.04 [-.06; .15]	-.27^***^ [-.36; -.18]	.85^***^ [.82; .88]	-					
(7) Perceived Acceptance by Teachers	3.64 (1.09)	.07 [-.03; .16]	.17^***^ [.08; .26]	.03 [-.07; .13]	-.33^***^ [-.41: -.24]	.44^***^ [.36; .52]	.58^***^ [.52; .64]	-				
(8) SAM Majority Face-to-Face	3.34 (1.15)	.06 [-.03; .16]	.38^***^ [.30; .46]	.18^***^ [.08; .27]	.14^**^ [.05; .24]	.20^***^ [.10; .28]	.17^***^ [.08; .26]	.18^***^ [.09; .27]	-			
(9) SAM Majority Online	2.42 (1.25)	-.07 [-.16; .03]	.31^***^ [.22; .39]	.11^*^ [.01; .21]	.16^***^ [.07; .25]	.07 [-.02; .17]	.07 [-.03; .16]	.13^*^ [.03; .22]	.54^***^ [.47; .60]	-		
(10) SAM Minority Face-to-Face	3.86 (0.98)	.27^***^ [.19; .36]	.18^***^ [.08; .27]	.10* [.003; .20]	.13^**^ [.04; .23]	.28^***^ [.19; .37]	.26^***^ [.17; .35]	.16^***^ [.07; .25]	.39^***^ [.31; .47]	.27^***^ [.18; .36]	-	
(11) SAM Minority Online	3.05 (1.28)	.15^**^ [.06; .25]	.18^***^ [.08; .27]	.10 [-.003; .20]	.12^*^ [.02; .21]	.08 [-.02; .17]	.08 [-.02; .17]	.05 [-.04; .15]	.22^***^ [.13; .31]	.57^***^ [.50; .63]	.56^***^ [.49; .62]	-
(12) Country of Origin Stereotyped	0.63 (0.48)	.13^*^ [.03; .22]	-.03 [-.13; .07]	.02 [-.08; .12]	.24^***^ [.14; .33]	.05 [-.04; .15]	.03 [-.07; .13]	-.11^*^ [-.20; -.01]	-.05 [-.15; .05]	-.16^**^ [-.25; -.06]	.07 [-.03; .17]	-.07 [-.16; .03]

Notes.^*^p < .05, ^**^p < .01, ^***^p < .001. Country of origin stereotyped was coded as 0 = non-negatively stereotyped, 1 = negatively stereotyped. SAM = Social Approach Motivation

### Indirect effects

To examine H1 – sense of belonging mediates the relationship of social identity threat and social approach motivation – we computed a path model with indirect effects including social identity threat as the predictor variable, sense of belonging to class as the mediator, and the four measures of social approach motivation (separate scales towards ethnic minority/ majority members, in face-to-face/ online settings) as the outcome variables. As a measure of social approach motivation towards ethnic minority members, we computed the mean of social approach motivation towards students from one’s own group of origin and social approach motivation towards students with other non-German backgrounds in the class, as 30% of the sample indicated that there were no other students with the same group of origin than themselves in their class. We therefore opted for averaging the items targeted towards students of one’s own culture of origin and students of other non-German groups of origin as the best approximation for social approach motivation towards ethnic minority members.

The path model was fully identified as all possible direct and indirect effects of social identity threat on the four measures of social approach motivation via sense of belonging were included. Results (Table 2) showed that social identity threat negatively predicted sense of belonging, which in turn positively predicted social approach motivation towards minority as well as majority members in face-to-face situations. The paths to social approach motivation towards minority and majority members in online situations were also positive. The indirect effects of social identity threat on face-to-face social approach motivation towards minority and majority members via sense of belonging were negative and significant. In contrast, the indirect effects on social approach motivation towards minority and majority members online were non-significant. In alternative models with sense of belonging to school and perceived acceptance by teachers as mediators, results were similar, but all direct and indirect effects were significant. Controlling for whether the countries of origin were negatively stereotyped in the German context did not change the results (except for the indirect effects on social approach motivation online being significant as well). Outputs of additional models can be found on the OSF.

**Table 2:** Results of Path Model Testing H1 with Sense of Belonging to Class as the Mediator

	*Direct Effects*														
	Sense of Belonging			SAM Minority Face-to-Face			SAM Minority Online			SAM Majority Face-to-Face			SAM Majority Online		
	*β* [95% CI]	*SE*	*p*	*β* [95% CI]	*SE*	*p*	*β* [95% CI]	*SE*	*p*	*β* [95% CI]	*SE*	*p*	*β* [95% CI]	*SE*	*p*
Social Identity Threat	- 0.18 [-0.27; − 0.08]	0.05	< .001	0.22 [0.13; 0.31]	0.05	< .001	0.18 [0.08; 0.27]	0.05	< .001	0.18 [0.09; 0.28]	0.05	< .001	0.18 [0.09; 0.27]	0.05	< .001
Sense of Belonging	--	--	--	0.32 [0.23; 0.40]	0.04	< .001	0.12 [0.02; 0.21]	0.05	.017	0.23 [0.14; 0.32]	0.05	< .001	0.10 [0.01; 0.20]	0.05	.031
	*Indirect Effects*														
	*β* [95% CI]	*SE*	*p*	*β* [95% CI]	*SE*	*p*	*β* [95% CI]	*SE*	*p*	*β* [95% CI]	*SE*	*p*	*β* [95% CI]	*SE*	*p*
via Sense of Belonging	--	--	--	-0.06 [-0.09; − 0.02]	0.02	.001	-0.02 [-0.04; 0.00]	0.01	.046	-.04 [-0.07; − 0.01]	0.02	.004	-0.02 [-0.04; 0.00]	0.01	.064

*Note*. Confidence intervals are displayed at the 95% level. SAM = Social Approach Motivation

### Moderated indirect effects

**Fig. 1:**
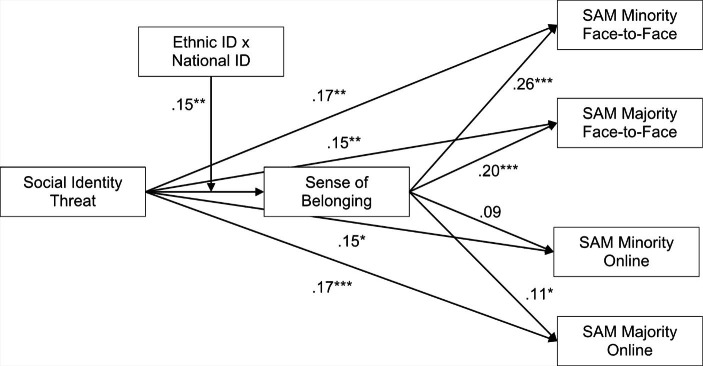
Results of the path model testing H2. Coefficients are standardized regression weights. Direct effects from ethnic and national identity are omitted. ^*^p < .05, ^**^p < .01, ^***^p < .001. SAM = Social Approach Motivation. ID = Identification.

To examine H2 – the interaction of ethnic and national identity moderates the relationship of social identity threat and sense of belonging – we computed a moderated mediation path model by introducing ethnic and national identity as well as all two-way interactions and the three-way interaction with social identity threat on sense of belonging as additional paths. All predictors involving the interactions were standardized. Fit indices for this model showed insufficient fit, χ²(24) = 133.92; *p* < .001, CFI = 0.94, TLI = 0.88, RMSEA = 0.10, SRMR = 0.09. Modification indices suggested that fit could be improved by including direct paths from ethnic identity and national identity to the four outcome variables of social approach motivation. These additional direct effects were included in a modified model, which showed good model fit (χ²(16) = 25.31; *p* = .065, CFI = 1.00, TLI = 0.99, RMSEA = 0.04, SRMR = 0.03). Results are displayed in Table 3; Fig. 1. The additional direct paths from ethnic and national identity on social approach motivation towards minority students in face-to-face and online contact situations were positive and significant (except for a non-significant path from ethnic identity to social approach motivation towards minority students online). National identity also positively predicted social approach motivation towards majority students in face-to-face and online situations. Ethnic identity was unrelated to social approach motivation towards majority students in face-to-face contact situations, but negatively related in online contact situations. Importantly and as expected, the hypothesized three-way interaction of social identity threat, ethnic identity, and national identity on sense of belonging was significant.

**Table 3:** Results of Path Model Testing H2 with Sense of Belonging to Class as the Mediator

	*Direct Effects*														
	Sense of Belonging			SAM Minority Face-to-Face			SAM Minority Online			SAM Majority Face-to-Face			SAM Majority Online		
	Coeff. [95% CI]	*SE*	*p*	Coeff. [95% CI]	*SE*	*p*	Coeff. [95% CI]	*SE*	*p*	Coeff. [95% CI]	*SE*	*p*	Coeff. [95% CI]	*SE*	*p*
X (Social Identity Threat)	-0.24 [-0.34; -0.15]	0.05	< .001	0.17 [0.09; 0.26]	0.04	< .001	0.15 [0.05; 0.24]	0.05	.002	0.15 [0.06; 0.23]	0.05	.001	0.17 [0.07; 0.26]	0.05	< .001
M (Sense of Belonging)	--	--	--	0.26 [0.18; 0.35]	0.04	< .001	0.09 [-0.002; 0.19]	0.05	.055	0.20 [0.11; 0.28]	0.05	< .001	0.11 [0.02; 0.20]	0.05	.019
W (Ethnic ID)	0.24 [0.15; 0.33]	0.05	< .001	0.10 [0.00; 0.18]	0.05	.045	-0.02 [-0.11; 0.08]	0.05	.698	-0.03 [-0.12; 0.06]	0.05	.558	-0.15 [-0.24; -0.06]	0.05	.002
Z (National ID)	0.06 [-0.04; 0.15]	0.05	.216	0.30 [0.22; 0.38]	0.04	< .001	0.25 [0.16; 0.34]	0.05	< .001	0.35 [0.27; 0.44]	0.04	< .001	0.30 [0.21; 0.38]	0.04	< .001
X x W	-0.004 [-0.10; 0.09]	0.05	.931	--	--	--	--	--	--	--	--	--	--	--	--
X x Z	-0.06 [-0.16; 0.05]	0.05	.269	--	--	--	--	--	--	--	--	--	--	--	--
W x Z	0.05 [-0.05; 0.14]	0.05	.327	--	--	--	--	--	--	--	--	--	--	--	--
X x W x Z	0.15 [0.04; 0.25]	0.05	.005	--	--	--	--	--	--	--	--	--	--	--	--
	*Conditional Indirect Effects*														
Low W Low Z	-0.013 [-0.06; 0.03]	0.02	.582	--	--	--	--	--	--	--	--	--	--	--	--
High W Low Z	-0.072 [-0.13; -0.02]	0.03	.010	--	--	--	--	--	--	--	--	--	--	--	--
Low W High Z	-0.094 [-0.16; − 0.03]	0.03	.004	--	--	--	--	--	--	--	--	--	--	--	--
High W High Z	-0.038 [-0.07; -0.003]	0.02	.033	--	--	--	--	--	--	--	--	--	--	--	--

*Note*. Confidence intervals are displayed at the 95% level. Coefficients of direct effects are standardized regression weights. SAM = Social Approach Motivation. ID = Identification.

**Fig. 2:**
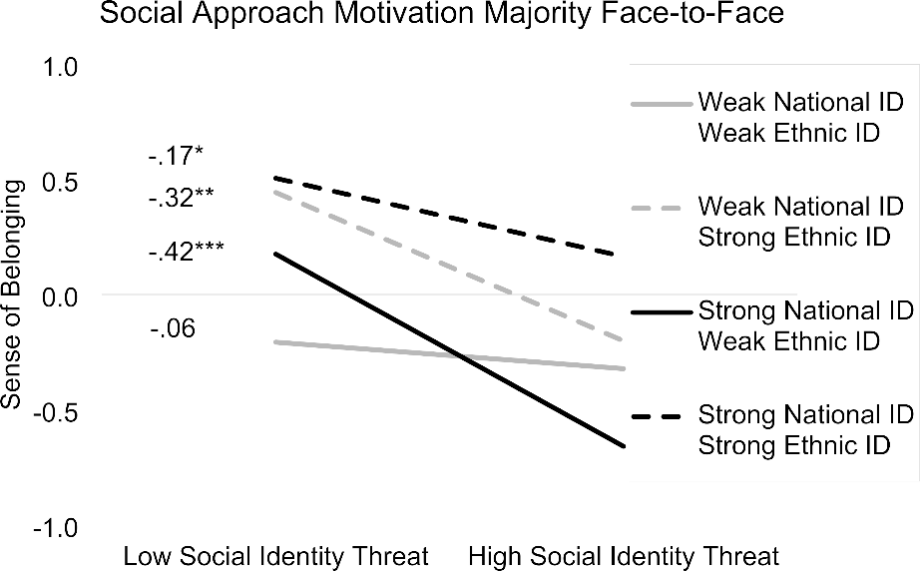
Simple slopes for the relationship of perceived social identity threat and sense of belonging at different levels of ethnic and national identity. Slopes are displayed at 1 SD below/above the mean (standardized). ^*^p < .05, ^**^p < .01, ^***^p < .001. ID = Identification

Simple slopes analyses (Fig. 2) revealed that the relationship of social identity threat and sense of belonging was non-significant for students with weak ethnic and weak national identity, but negative and significant for all other student groups. Correspondingly, and indicating moderated mediation, for social approach motivation towards majority members in face-to-face situations the conditional indirect effects were non-significant for students with weak ethnic and weak national identity, but negative and significant for students endorsing one identity strongly and the other one weakly. Contrary to expectations, the indirect effect for students with high ethnic and high national identity was also significant, albeit smaller in size than the indirect effects for students endorsing one identity strongly and the other identity weakly. For social approach motivation towards minority members in face-to-face situations, the three-way interaction was also significant and conditional indirect effects were very similar. In line with results for H1, for social approach motivation towards minority and majority members in online situations, the three-way interaction was significant, but the conditional indirect effects were not. Again, alternative models with sense of belonging to school and perceived acceptance by teachers as mediators showed comparable results. Controlling for whether the countries of origin were negatively stereotyped in the German context did not change the results. Outputs of additional models can be found on the OSF.

## Discussion

The current research investigated the indirect relationship of social identity threat with social approach motivation for ethnic minority adolescents in German secondary schools. Results replicated previous findings with ethnic minority adults in Norway showing an indirect effect of social identity threat on social approach motivation via reduced sense of belonging (Froehlich et al., 2023). In addition, the current research went beyond previous studies and investigated the generalizability of (moderated) indirect effects for different measures of sense of belonging and social approach motivation.

### Lower social approach motivation towards all classmates, but mainly in face-to-face situations

Concerning group membership of classmates, higher perceptions of social identity threat were indirectly associated with lower social approach motivation towards both ethnic minority and majority classmates via reduced sense of belonging. In contrast to ethnic homophily (Branscombe et al., 1999; Leszczensky & Pink, 2019), perceptions of social identity threat seem to go along with general feelings of not belonging at school/in class, which in turn impair ethnic minority students’ motivation to make friends among German and ethnic minority classmates alike. This is concerning, as it puts ethnic minority students experiencing social identity threat at risk of social isolation, which potentially contributes to ethnic performance disparities in the German educational system (Raabe, 2019; Stanat et al., 2019; Zander, 2022). A potential explanation for this generalized effect on social approach motivation might be based on the multi-threat framework (Shapiro & Neuberg, 2007), which describes different kinds of social identity threat. For ethnic minority students, who are highly identified with their ethnic group and who are not very likely to personally endorse the stereotype about their ingroup’s low academic ability, Shapiro (2011) showed that they tend to experience (combinations of) own-reputation threat (i.e., the fear of stereotypic characterization of the self in the eyes of ingroup or outgroup others) and group-reputation threat (i.e., the fear of reinforcing negative stereotypes about one’s group in the minds of ingroup or outgroup others). If both ethnic minority and majority members are potential sources of social identity threat, this might explain generalized withdrawal from classmates in the academic context. As ethnic identity was comparatively high in the current sample, in line with the rejection-identification model ethnic minority students might reinforce their threatened identity with ethnic ingroup members outside of the academic domain (e.g., family members, peers in extracurricular or leisure activities). In order to shed light on these potentially complex strategies to cope with social identity threat, future research should include more detailed measures of different types of social identity threat as well as measures of social approach motivation towards ethnic ingroup and outgroup members inside and outside of school.

Concerning the mode of communication, results were specific to social approach motivation in face-to-face situations. In online situations, the (moderated) indirect effect was not significant. Although online interactions with peers are frequent (Hamilton et al., 2022; Twenge et al., 2019), our results indicate that motivation to approach others in face-to-face situations is more prone to be affected by worries about negative stereotypes. Because adolescents’ online and offline social networks are largely overlapping (Liu et al., 2016; Reich et al., 2012) and students usually spend most of their time with peers in class in face-to-face interactions, this type of contact seems to be the more central outcome in the context of the present research. However, results should be interpreted with caution as we only assessed the strength of social approach motivation, but not the frequency of contact in face-to-face vs. online situations. Therefore, with the current data it is not possible to discern whether contact in online situations is more infrequent or is affected by social identity threat to a lesser degree than contact in face-to-face situations. Moreover, face-to-face contact might have been more salient than online contact, as students filled out the questionnaires in their classrooms in the presence of their classmates.

Concerning the different levels of sense of belonging, contrary to expectations, results were more pronounced for sense of belonging to school and perceived acceptance by teachers compared to sense of belonging to class. This might be explained by the fact that the class mainly consists of ethnically heterogeneous peers with equal status, whereas higher-status teachers as well as other staff at the general academic institution are predominantly ethnic majority members. Ethnic minority students might perceive these higher-status majority members as sources of social identity threat (Shapiro & Neuberg, 2007). Therefore, perceived acceptance by teachers and membership in the school community might be more central mediators for the relationship of social identity threat and social approach motivation. In line with this, Celeste et al. (2019) showed that ethnic gaps in sense of belonging and achievement were smaller in schools with school policies emphasizing multiculturalism (i.e., embracing cultural diversity) vs. assimilation and colorblindness (i.e., rejecting or ignoring diversity). Similarly, Schachner et al. (2019) showed that school policies of cultural pluralism (embracing cultural diversity as a resource) as well as equality and inclusion (promoting positive intergroup contact) positively predicted achievement of all students via increased sense of belonging. Therefore, policies embracing cultural diversity and intergroup contact on the school level seem to be more beneficial for students’ sense of belonging and performance than ignoring or rejecting diversity. Such multicultural policies might also have the potential to increase minority students’ perceived acceptance by teachers and decrease social identity threat.

### How ethnic minority adolescents construct their multiple social identities

Similar to previous research (e.g., Froehlich et al., 2020; Martiny et al., 2017; Martiny et al., 2020), ethnic identity was endorsed more strongly than national identity with dual identity falling in between. Ethnic and national identity were unrelated, whereas dual and national identity were positively related. According to Fleischmann and Phalet (2016), these results can be interpreted as ethnic and national identity being compartmentalized (i.e., endorsement of one identity being independent and disassociated from endorsement of the other identity), but dual and national identity being compatible or overlapping (i.e., higher endorsement of one identity goes along with higher endorsement of the other identity). Although in the current ethnically heterogeneous sample we did not find the negative relation between ethnic and national identity shown for example in a previous study with Turkish-origin migrants attending school in Germany (Martiny et al., 2017), results again point into the direction that ethnic minorities construct their multiple social identities in a way creating the least identity conflict (Amiot et al., 2007; Benet-Martínez, 2012; Hirsh & Kang, 2016). Dual identity seems to be more compatible with national than ethnic identity in the assimilationist context of Germany (Benet‐Martínez, 2012). However, not only the levels and interrelations of multiple social identities are relevant, but also their consequences for social relationships in the academic context.

### The interplay of multiple social identities, social identity threat and sense of belonging in the social domain

In line with Froehlich et al. (2023), the interplay of ethnic and national identity moderated the magnitude of the indirect effect of social identity threat on social approach motivation. Similar to the study with university students in Norway (Froehlich et al., 2023), there was a negative relationship of social identity threat and sense of belonging for students with an identity pattern reflecting the acculturation orientation of separation (i.e., strong orientation towards the culture of origin and weak orientation towards the national culture; Berry 1997). As the ethnic group is the target of negative stereotypes, it is expected that these students are susceptible to the consequences of social identity threat in the social domain in terms of impaired social approach motivation.

In turn, results for the other combinations of ethnic and national identity were partly not in the expected direction. First, students with the acculturation orientation of assimilation (i.e., strong orientation towards the national culture and weak orientation towards the culture of origin) were also susceptible to the consequences of social identity threat in the social domain. Only identifying with the cultural group of Germans and not with the ethnic culture of origin might represent a strategy of social mobility (i.e., psychologically leaving the negatively stereotyped ingroup; Tajfel & Turner 1979) in an attempt to attenuate the effects of social identity threat that might be chronically and cumulatively active in academic contexts (Steele, 1997; Steele et al., 2002). An assimilation orientation was shown to be beneficial for academic performance in the German educational context (e.g., Hannover et al., 2013). However, for social motivation instead of performance, this individual mobility strategy of leaving one’s ingroup and joining the majority group does not seem to be effective, as ethnic group boundaries are usually impermeable and thus the attempt at “passing for a majority group member” is not successful. This might the case because ethnic minority members’ self-identification (feeling German) often does not align with majority members’ perceptions of their ethnicity (being categorized as an ethnic minority member; “identity denial”; e.g., Boda & Néray 2015; Verkuyten, 2018). Social approach motivation was lower for these students as well, which might be explained by this potential mismatch between self-identification and ethnic categorization by majority members.

In contrast to findings from Froehlich et al. (2023), the relationship of social identity threat and sense of belonging was non-significant for students with an acculturation orientation of marginalization (i.e., not identifying with either ethnic group). For these students, sense of belonging seems to be relatively low in general, but multiple social identities related to ethnic groups were no relevant predictors. Other factors related to personal identities or social identities on other demographic dimensions (e.g., Hannover & Zander 2020; Zander & Hannover, 2014) might be more relevant for students disengaged from ethno-cultural identities to predict social approach motivation and the choice of academic peer friendships.

Finally, contrary to our hypotheses, the relationship of social identity threat and sense of belonging was also negative and significant for students showing an acculturation orientation of integration (i.e., strong orientation towards the national and the ethnic group of origin). However, the effect size was smaller than for students endorsing one identity strongly and the other one weakly. Compared to results from Froehlich et al. (2023) with adults, the buffering effect of integrated multiple social identities was less pronounced for adolescents. One possible explanation is that dual identity is still developing during adolescence. For example, a longitudinal study with Muslim minority adolescents in Germany showed that dual identity development continued throughout late adolescence (Spiegler et al., 2019).

### Practical implications

Results of the current study have implications for schools and educational practice. There are several routes through which ethnic minority students can be supported in order to reduce ethnic disparities based on stereotypes and stereotype threat. One route would be to decrease the salience of ethnic group memberships and the activation of ethnic stereotypes in the school context. For example, teachers could contribute to blurring ethnic group boundaries by creating a common ingroup identity among students for example as members of the same school or classroom (Gaertner et al., 2008). Second, interventions can reduce ethnic minority students’ susceptibility to stereotype threat, for example by affirming multiple social identities or by informing students about stereotype threat and its detrimental consequences (Liu et al., 2021). In addition, teachers can implement collaborative learning activities that foster intergroup contact, mutual interdependence, and cooperation like the jigsaw puzzle method (Aronson et al., 1978) in order to bring students from different ethnic backgrounds into contact and to reduce the social isolation of minority students who feel threatened by stereotypes. Finally, creating a class and school climate emphasizing the value of diversity can support not only minority students, but is beneficial for all students (e.g., Schachner et al., 2019).

### Limitations and future directions

A first limitation of the current study is that it is cross-sectional and therefore no conclusions about causal relationships between variables can be drawn. Future research should therefore include longitudinal measurements of social identity threat, sense of belonging, and social approach motivation as well as experimental manipulations of social identity threat to corroborate the directionality of results. Second, the current study only included self-report measures of social approach motivation, but not of behavioral tendencies or actual behavior in social situations. Building on results from Froehlich et al. (2023) who showed that social approach motivation predicted behavioral intentions to attend social events, future research should incorporate actual behavioral measures or social network analysis (e.g., Leszczensky & Stark 2020) to further investigate the relevance of social identity threat for ethnic segregation of peer networks. Relatedly, the current research did not include measures of social avoidance motivation and academic performance. As impaired social approach motivation might contribute to ethnic disparities in educational success, future studies should jointly investigate the consequences of social identity threat for performance and the social domain. Social avoidance motivation (i.e., the motivation to avoid negative social outcomes like social rejection; Nikitin & Schoch 2021) or social anxiety might also potentially play a role for the current results, for example concerning the weaker effects in online compared to face-to-face contact situations. Previous research did not consistently find associations of social identity threat and social avoidance motivation (Martiny & Nikitin, 2019; Rahn et al., 2021), however, a potential role of social avoidance motivation cannot be ruled out in the current research.

Moreover, we interpreted the associations between ethnic, national, and dual identities as compatibility, conflict, and compartmentalization (Fleischmann & Phalet, 2016). Due to questionnaire length, a direct measure of identity conflict and compatibility (e.g., Benet-Martínez & Haritatos 2005) was not included. Furthermore, the fact that differing questionnaire versions were available for different patterns of ethnic self-identification might have made ethnic group membership and associated academic stereotypes salient at the beginning of the study. We conducted a detailed debriefing discussing the possibility that stereotypes might give rise to negative thoughts and emotions and how to deal with them (see codebook for full debriefing). Nevertheless, it cannot be ruled out that the procedure might have influenced students’ sense of belonging and social approach motivation.

Lastly, whereas the indirect effect of social identity threat on social approach motivation via reduced sense of belonging has been replicated for different target groups (gender, ethnicity, age), contexts (university, high school, the workplace) and methods (experiments and surveys), results for the direct effect of social identity threat on social approach motivation were mixed with sometimes negative and sometimes positive relationships (Froehlich et al., 2023; Martiny & Nikitin, 2019; Rahn et al., 2021). As the indirect effect via sense of belonging does not fully account for the association between social identity threat and social approach motivation, it is likely that other mechanisms are additionally at play. For example, a positive association of social identity threat and social approach motivation might be due to collective action intentions in an attempt to refute the stereotype by approaching others or due to a desire to reconnect with others after experiencing social identity threat (e.g., DeWall & Richman 2011). Future research should therefore explore alternative mediators to shed light on potential parallel mechanisms and investigate which mechanisms are dominant for which target individuals and contexts.

## Conclusion

Peer relationships are relevant for the educational success of ethnic minority students from negatively stereotyped countries of origin (Raabe, 2019), who underperform compared to German students (Stanat et al., 2019). The current study showed that worries about being evaluated in light of negative stereotypes about one’s ethnic group has consequences for ethnic minority members’ motivation to approach academic peers already in adolescence. To attenuate the risk of social isolation, interventions to reduce feelings of social identity threat (Liu et al., 2021) as well as to affirm dual identities (Celeste et al., 2021) should be implemented in ethnically diverse secondary schools.

## Footnotes

1 The following additionally assessed constructs are not reported in this manuscript: perceived integration (2 items; Martiny et al., 2017), acculturation orientations (20 items, Maehler 2018), intergroup contact (3 items; Martiny et al., 2017), domain identification (2 items, Keller 2007), socioeconomic status (Mang et al., 2019), and subjective socioeconomic status (Adler et al., 2000).
